# An increasing number of states filled Conrad 30 waivers for recruiting international medical graduates

**DOI:** 10.1093/haschl/qxae103

**Published:** 2024-08-19

**Authors:** Tarun Ramesh, Sarah E Brotherton, Gregory D Wozniak, Hao Yu

**Affiliations:** Department of Population Medicine, Harvard Medical School and Harvard Pilgrim Health Care Institute, Landmark Center, 401 Park Drive, Suite 401 East, Boston, MA 02215, United States; Health Outcome Analytics, American Medical Association, Chicago, IL 60611, United States; Health Outcome Analytics, American Medical Association, Chicago, IL 60611, United States; Department of Population Medicine, Harvard Medical School and Harvard Pilgrim Health Care Institute, Landmark Center, 401 Park Drive, Suite 401 East, Boston, MA 02215, United States

**Keywords:** health policy, international medical graduates, Conrad 30

## Abstract

To address physician shortages in the United States, Congress created the Conrad 30 visa waiver program allowing non-citizen international medical graduates to obtain visas to practice medicine in underserved areas. There is little information on whether states have effectively used the program. To fill the gap, we examined the growth and distribution of Conrad physicians between 2001 and 2020. We found that the number of states filling all of their annual allocated Conrad slots increased over the last two decades, yet one-half of the states still did not fill their allowed slots in 2020. Our analysis also revealed substantial variations across states in the number of Conrad physicians by specialty (eg, primary care physicians and psychiatrists), geography (eg, rural vs urban areas and physician shortage vs non-shortage areas). Our findings suggest that states can better use the Conrad program to meet healthcare needs across specialties and geographic areas.

## Introduction

With increased retirements due to an aging workforce and provider burnout exacerbated by the COVID-19 pandemic, the physician workforce supply continues to lag behind healthcare demand care.^1,2^ Unmet healthcare needs in the Health Resources and Services Administration (HRSA) designated Health Professional Shortage Areas (HPSAs) have translated to reduced utilization of preventive care, increased all-cause mortality, and higher COVID-19-related mortality.^3-6^

Efforts to combat the physician shortage include increasing funding for the National Health Service Corps (NHSC), expanding scope of practice of Advanced Care Practitioners, and implementing the Conrad 30 Waiver program, which offers an important path for some international medical graduates (IMGs) to bolster the domestic physician workforce.^7^ In 2019, IMGs composed 24.7% of all active physicians.^8^ Previous research found that IMGs were likely to practice as primary care physicians (PCPs) in rural areas and that overall health outcomes were similar between IMGs and US medical graduates.^9-12^ However, the role of non-citizen IMGs in addressing physician shortages is less studied.

Non-citizen IMGs can legally train in US medical residency programs through the H-1B and J-1 visa pathways.^13^ Since H-1B visas are sponsored by individual training hospitals with a filing fee, many hospitals choose not to sponsor visas.^14-16^ In comparison, the vast majority of non-citizen IMGs enter the country through the J-1 visa pathway sponsored by the Educational Commission for Foreign Medical Graduates with no fees for individual training programs.^17^ The Immigration and Nationality Act requires those J1 trainees upon completion of their postgraduate medical training to return to their country of origin for 2 years. During that time, they may apply for a traditional immigrant visa (eg, H-1B) to return to the United States. However, the Conrad program waives the requirement by allowing those trainees to convert their J-1 and spouse/child J-2 visa to H-1B nonimmigrant status in exchange for a 3-year full-time employment contract to practice medicine in underserved areas, such as in HPSAs and Medically Underserved Areas, as designated by the HRSA.^18,19^ Approximately 3500 IMGs were on H-1B visas in 2008, compared with more IMGs (6100) on J1 visas in the same time.^17^

The Conrad program is both an important pathway for IMGs with J-1 visas to enter the US workforce and a large physician recruitment program targeting shortage areas.^20^ The 1994 Immigration and Nationality Technical Corrections Act allowed each state to sponsor up to 20 Conrad waivers each year.^21^ In 2003, Congress increased the cap to 30 per state per year. Eligibility criteria and application requirements vary by state, such as qualifying specialties and practice areas.^22^ States are able to determine qualifying specialties for Conrad waivers and the number of slots for a qualified specialty within a state. For example, California restricted Conrad 30 waiver spots only to PCPs until 2023, when the state broadened eligible applicants to non-PCPs as well.^23^ Additionally, the Conrad program allows states to use 10 flex spots among the annual 30 slots to place physicians in non-shortage areas. Previous research has analyzed the overall distribution of physicians recruited through the Conrad waiver program and found that lower percentages of primary care and rural physicians were recruited through the program over time.^24^

However, there is little information on the state-level trends in growth and distribution of Conrad physicians by specialty (eg, primary care), geography (eg, rural areas), and type of spots (eg, flex spots). This study aimed to fill the gap by using 20-year data.

## Data and methods

This retrospective analysis employed repeated cross-sectional data, which were publicly available without personal identifiers, and our study was determined non-human subjects research by the corresponding author's institutional review board. Our study followed the STROBE reporting guideline for cross-sectional studies.

We used annual data from the Rural Recruitment and Retention Network on Conrad waiver spots filled by each state in 2001-2020. The Rural Recruitment and Retention Network supports healthcare workforce development in rural and underserved areas and collects annual data from state Conrad 30 program administration offices. We merged the data by state and year with HPSA populations.^25^ We assessed trends in the growth and distribution of Conrad physicians by geography and specialty. The Conrad program defines PCPs as those completing residencies in family medicine, internal medicine, pediatrics, or obstetrics and gynecology.

### Limitations

This study has some limitations. First, it did not evaluate Conrad physician retention in communities in which they are placed. Second, it did not have information about physician practice locations below the state level. Third, the data include the number of flex spots since 2006 and the number of psychiatrists from 2018, but no information about other specialists (eg, anesthesiologists and cardiologists) that the program supported. Finally, we did not evaluate the impacts of Conrad physicians on healthcare or health outcomes. Improved data infrastructure can help with further evaluations of the Conrad program effects.

## Results

### Distributions of Conrad physicians across states

**Figure 1.**
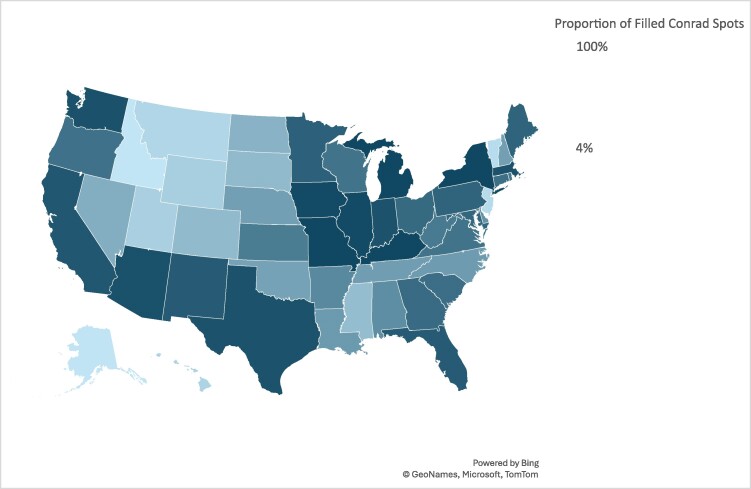
Proportion of filled Conrad waiver spots by states from 2001 to 2020. Authors’ analysis of the Rural Recruitment and Retention Network data.

The Conrad program recruited 18 504 physicians from 2001 to 2020, but the distribution varied substantially across states. Figure 1 indicates that there were fewer physicians in Southern and Great Plains states and more in states clustered in the Midwest and Northeast.

**Figure 2.**
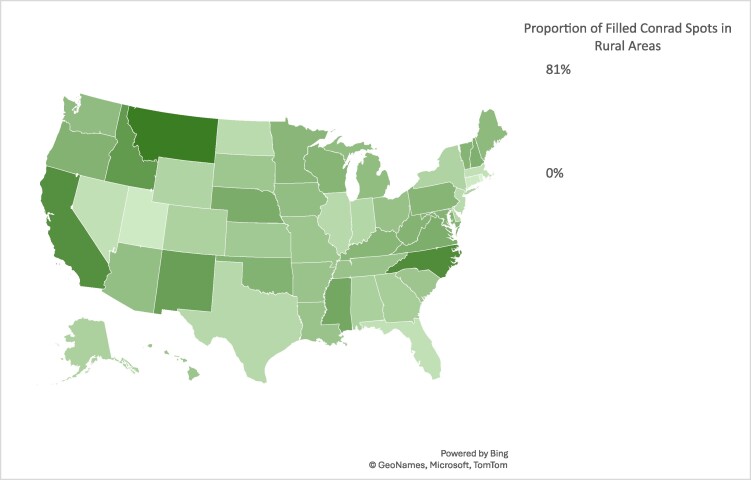
Proportion of filled Conrad waiver spots in rural areas by states from 2004 to 2020. Authors’ analysis of the Rural Recruitment and Retention Network data.

Figure 1 indicates substantial variations in the number and proportion of filled Conrad physicians across states in 2001-2020. Three states (Kentucky, Michigan, and New York) filled all the allowed Conrad slots (590) throughout the study period, while Idaho (23), Alaska (26), Vermont (54), and New Jersey (60) filled the fewest. On average, all the states and Washington, DC, filled 61% of all the allowed Conrad slots in the study period with 12 states (Arizona, California, Illinois, Indiana, Iowa, Kentucky, Massachusetts, Michigan, Missouri, New York, Texas, and Washington) reaching 90% or higher, compared with four states at 10% or lower (Alaska, 4%; Idaho, 4%; New Jersey, 10%; and Vermont, 9%). While three of these states (Alaska, Idaho and Vermont) have relatively small populations, it is worth noting higher proportions of filled Conrad slots in other states with small populations, such as Maine (83%) and Rhode Island (71%). Figure 2 shows that the states differed considerably in the proportion of Conrad physicians in rural areas. Six states (Idaho, 61%; Mississippi, 51%; New Mexico, 57%; California, 67%; North Carolina, 70%; Montana, 81%) recruited more than half of their Conrad physicians to rural areas, compared with <10% in three states (Connecticut, 6%; Rhode Island, 0.2%; and Utah, 6%) (See Appendix for additional information).

**Figure 3.**
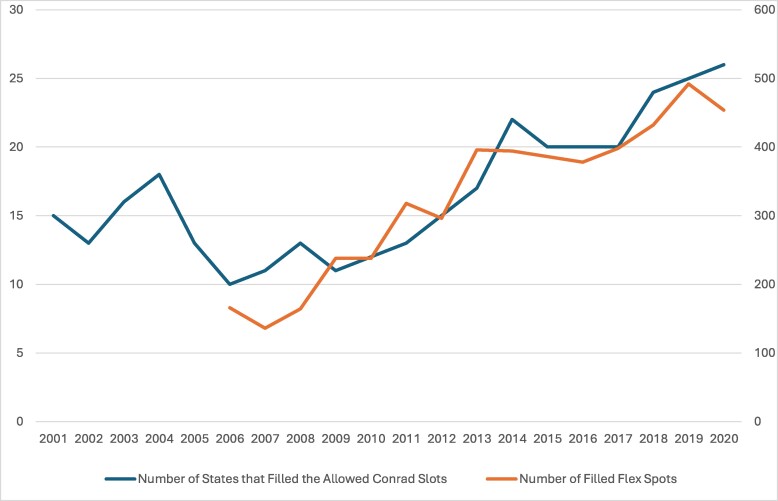
Number of states that filled the allowed Conrad slots by year and the growth of flex waiver spots utilized in the Conrad 30 waiver program. Authors’ analysis of the Rural Recruitment and Retention Network data.

Figure 3 shows that the number of states (including Washington, DC) that filled all the Conrad slots allowed by Congress increased markedly from 15 in 2001 to 26 in 2020 and that half of states still did not fill all the allocated slots in 2020.

The Conrad program recruited 8302 PCPs in 2001-2020, accounting for 45% of all Conrad physicians (Table 1). California recruited the most PCPs (485) in 2001-2020. In 2020, California filled every Conrad slot with PCPs. Regarding the ratio of Conrad PCPs per 100 000 Primary Care HPSA population in 2020, New Jersey (73.09), New Hampshire (4.24), and Maine (3.17) had the highest. However, the total HPSA populations in these states are much lower than other states such as Texas, Kentucky, and Tennessee. Three states (Oklahoma, Nebraska, and Utah) did not recruit any PCPs in 2020 despite their substantial populations in HPSA areas (Appendix Table S1).

**Table 1.** Distribution of Conrad physicians by states and specialty.

State	PCPs as a proportion of all Conrad physicians (2001-2020) (%)	Number of Conrad PCPs per 100 000 primary care HPSA population (2020)	Number of Conrad psychiatrists per 1 000 000 MHPSA population (2020)
Alabama	21	0.09	0.68
Alaska	12	0.00	0.00
Arizona	60	0.35	0.70
Arkansas	21	0.31	0.00
California	90	0.36	0.29
Colorado	47	0.80	0.00
Connecticut	52	1.36	4.45
Delaware	39	1.46	7.47
Dist. of Col	65	2.91	0.00
Florida	65	0.10	0.31
Georgia	53	0.39	0.20
Hawaii	32	0.20	0.00
Idaho	87	0.00	0.00
Illinois	51	0.39	0.58
Indiana	44	0.42	0.90
Iowa	37	1.20	0.55
Kansas	22	0.64	0.00
Kentucky	41	0.07	0.00
Louisiana	56	0.40	0.00
Maine	31	3.17	0.00
Maryland	47	1.62	0.78
Massachusetts	51	1.88	10.98
Michigan	49	0.39	0.95
Minnesota	43	1.02	0.56
Mississippi	42	0.00	0.00
Missouri	33	0.18	0.00
Montana	26	0.24	0.00
Nebraska	23	0.00	0.00
Nevada	62	0.21	0.41
New Hampshire	45	4.24	10.74
New Jersey	73	73.09	25.18
New Mexico	38	0.47	0.73
New York	50	0.28	1.71
North Carolina	67	0.87	1.20
North Dakota	25	0.53	3.30
Ohio	35	0.34	0.84
Oklahoma	25	0.00	0.00
Oregon	66	1.64	0.00
Pennsylvania	39	0.20	0.58
Rhode Island	31	3.13	7.08
South Carolina	45	0.58	0.00
South Dakota	27	1.18	0.00
Tennessee	31	0.04	0.00
Texas	32	0.08	0.13
Utah	48	0.00	0.00
Vermont	26	0.00	
Virginia	54	0.37	1.54
Washington	57	0.34	0.71
West Virginia	33	0.77	0.00
Wisconsin	56	0.62	0.91
Wyoming	16	0.53	0.00
United States	45	0.41	0.69

Authors’ analysis of the Rural Recruitment and Retention Network data.

Similarly, there were substantial variations in the number of Conrad psychiatrists recruited across states in 2018-2020 (Table 1). New York (25), Connecticut (15), and Massachusetts (12) recruited the most, while 11 states did not recruit any psychiatrists from 2018 to 2020. In 2020, New Jersey, Massachusetts, and New Hampshire had the highest ratio of Conrad psychiatrists per 100 000 Mental Health HPSA population (Table 1). Overall, the total number of psychiatrists was small (203) in 2018-2020, and the number remained steady in 2018 (69), 2019 (69), and 2020 (65).

### Filled flex spots

The number of filled flex spots grew from 166 in 2006 to 454 in 2020—a 173% increase (Figure 3). States with the greatest number of filled flex spots included Kentucky (130) and North Dakota (128); these two states (Kentucky, 87%; North Dakota, 85%) ranked the highest in terms of the filled flex spots as a proportion of the allowed flex spots. Some states, including Arizona and Missouri, did not use any flex spots. Vermont (78%) and Wyoming (70%) ranked the highest in the number of flex spots as a proportion total Conrad physicians recruited (Table 2).

**Table 2.** Number of filled flex spots by States.

State	Filled flex spots (2006-2020)	Filled flex spots as % of the allowed flex spots (2006-2020)	Filled flex spots as % of all Conrad physicians (2006-2020)
Alabama	6	4	2
Alaska	5	3	19
Arizona	0	0	0
Arkansas	78	52	22
California	3	2	1
Colorado	52	35	29
Connecticut	6	4	1
Delaware	12	8	4
Dist. of Col	18	12	13
Florida	12	8	2
Georgia	32	21	7
Hawaii	53	35	61
Idaho	13	9	57
Illinois	12	8	2
Indiana	21	14	4
Iowa	88	59	15
Kansas	25	17	6
Kentucky	130	87	22
Louisiana	53	35	18
Maine	85	57	17
Maryland	92	61	19
Massachusetts	34	23	6
Michigan	24	16	4
Minnesota	106	71	21
Mississippi	18	12	11
Missouri	0	0	0
Montana	20	13	26
Nebraska	102	68	37
Nevada	9	6	4
New Hampshire	86	57	31
New Jersey	18	12	30
New Mexico	101	67	19
New York	25	17	4
North Carolina	66	44	23
North Dakota	128	85	62
Ohio	107	71	23
Oklahoma	69	46	26
Oregon	34	23	8
Pennsylvania	42	28	9
Rhode Island	50	33	12
South Carolina	65	43	14
South Dakota	106	71	59
Tennessee	24	16	9
Texas	1	1	0
Utah	40	27	40
Vermont	42	28	78
Virginia	59	39	14
Washington	25	17	5
West Virginia	88	59	20
Wisconsin	84	56	19
Wyoming	73	49	70
United States	2443	32	13

Authors’ analysis of the Rural Recruitment and Retention Network data.

## Discussion

This study provides important information about states’ implementation of the Conrad program over the past two decades. While the number of states filling all the allowed Conrad slots increased remarkably over the study period, half of the states still did not fill their allowed slots in 2020, highlighting state-level deficits in the program uptake. Whereas three states (Kentucky, Michigan, and New York) filled every slot in each study year, others (Alaska, Idaho, New Jersey, and Vermont) did not fill all the allowed slots in any year. The unfilled slots may be due to barriers to physician recruitment, including physician-community mismatches, lack of funding or staff, burdensome waiver application process (which varies by state), and inadequate technical assistance from federal agencies, as noted by a qualitative study.^19^

There are considerable variations across states in the number of Conrad PCPs, with some states recruiting either no or few PCPs despite substantial primary care HPSA populations. These low levels of PCP recruitment may be due to multiple factors, such as the reductions in physicians pursuing primary care, difficulty in physician recruiting overall, and lack of specific recruitment efforts targeting PCPs. States also differed greatly in the proportion of Conrad physicians in rural areas. For some of these states (ie, Rhode Island, 0.2%; New Jersey, 17%), this is likely due to the limited rural primary care HPSA areas within the state. For other states (eg, Utah, 6%), it is concerning to have such a low proportion of rural Conrad physicians when the state is largely rural. Future studies need to understand why states like Utah were not able to recruit more Conrad physicians to their rural areas.

The flex spots which allow states to place Conrad physicians in non-shortage areas could help states recruit much needed non-primary care specialists, including surgeons and other specialists. However, overreliance on flex spots could unintentionally disincentivize physicians to work in shortage areas. For example, North Dakota had the largest number of the filled flex spots, while having a very low proportion of Conrad physician recruited to rural areas. Future research needs to evaluate how states, especially rural states, maintain a balance between targeting physician shortage areas through the conventional Conrad program and recruiting Conrad physicians to non-shortage areas using the flex spots.

Our study results have several important policy implications. The unequal distribution of the Conrad physicians across states suggests there are significant state-level barriers to recruitment. State policymakers must recognize the program's substantial public health benefit and make efforts to recruit a steadier stream of Conrad physicians to underserved areas year after year. Federal agencies should encourage states with high HPSA populations to make use of the program through technical and financial support. Additionally, coordinating federal and state roles and responsibilities to improve the program should be a priority. For example, immigration barriers such as long waiting times and technical assistance in visa applications should be referred to relevant federal agencies, while states should focus their resources on identifying communities with high need and targeting those areas in their recruitment efforts. The program has the potential to bolster the physician workforce in shortage areas, but intensified recruitment and sustainable retention within these areas are key. Further analysis and funding should prioritize continuous care in communities with limited access to providers. Both the Conrad program and the NHSC aim to attract clinicians to underserved areas, but these two programs differ from each other in terms of clinician types and incentives. While the Conrad program focuses exclusively on recruiting international physicians by helping them obtain H-1B visas after they complete residency training in the United States, the NHSC program offers scholarships and loan repayments to attract physicians and non-physician clinicians, both of whom are required to have US citizenship or nationality. Additionally, the NHSC targets exclusively HPSAs, while the Conrad program may recruit physicians to HPSAs or Medically Underserved Areas with the additional option for states to use the “flex slots” to recruit Conrad physicians to those areas that is not HPSAs or Medically Underserved Areas. Because of its cap of 30 slots per state per year, the Conrad program has a limited recruitment capacity with <20 000 physicians recruited over the past two decades. In comparison, ∼41 180 clinicians, the majority of whom are non-physician clinicians, were recruited by the NHSC during the same period.^26^ The differences between the two programs offer insight into how to best improve clinician recruitment in underserved areas.

A 2006 Government Accountability Office survey of state officials in all the 50 states and Washington, DC, found that 80% of the survey respondents felt that the 30-waiver limit was adequate.^20^ Since that survey, 16 years have passed. Our finding that 26 states filled all their allowed slots in 2020, compared with only 15 in 2001, suggests that further evaluation may be needed to determine whether states have the capacity to host additional slots to address the growing health professional shortages, including both primary care and mental health professional shortages.^27,28^ Moving away from an allocation process that distributes available spots equally across states to one that is proportionate to population or unmet clinical need could help support states with great need but few recruits. A previous qualitative interview of Conard program staff identified several obstacles to recruitment and retainment of physicians in rural communities, including lack of funding for program oversight, legal fees for waiver processing, and difficulty recruiting to high-poverty communities.^20^ Future studies need to assess whether states are attempting to use all 30 of their annual waivers and why some states are not able to fill all the 30 slots allowed annually.

Additional research also needs to understand why states with relatively large population filled few Conrad slots (eg, New Jersey, which ranks 11th by population, filled 10% of Conrad slots in 2001-2020). Future studies also need to examine how the Conrad program may be affected by a recent Tennessee law for recruiting IMGs by offering them a new “provisional licensing” pathway to attain fully licensed physician status, especially given similar bills pending in other states.^29^ This new Tennessee law, which has been modeled by other states (Florida, Missouri), would permit IMGs who have previously practiced in their origin country to obtain US medical licensure after a supervision period without having to graduate from a US residency or fellowship program. These laws aim to lower barriers for skilled immigrants and to improve care access in underserved and rural areas. However, both the Conrad program and state provisional licensing pathways have medical “brain drain’ implications for the global health workforce that should be further studied.

## Conclusions

There has been a substantial growth of Conrad physicians during the past two decades with an unequal distribution of those physicians across states and by specialty, geography, and type of spots. Both federal and state policymakers need to better coordinate their efforts to make the Conrad program more effective for improving physician supply in underserved areas. They also need to better use the Conrad program to meet healthcare needs across specialties (eg, primary care vs other specialties), between rural and urban areas, and between physician shortage and non-shortage areas through filling both the conventional and flex Conrad program spots.

## Supplementary Material

qxae103_Supplementary_Data

## References

[1] Bhardwaj A . COVID-19 pandemic and physician burnout: ramifications for healthcare workforce in the United States. J Healthc Leadersh. 2022;14:91–97. 10.2147/JHL.S360163 35726282 PMC9206033

[2] Impact of the COVID-19 pandemic on the hospital and outpatient clinician workforce: challenges and policy responses (Issue Brief No. HP-2022-13). Office of the Assistant Secretary for Planning and Evaluation, U.S. Department of Health and Human Services; May 2022. Accessed August 5, 2023. https://aspe.hhs.gov/reports/covid-19-health-care-workforce

[3] Brown TM , Parmar G, Durant RW, et al Health professional shortage areas, insurance status, and cardiovascular disease prevention in the Reasons for Geographic and Racial Differences in Stroke (REGARDS) study. J Health Care Poor Underserved. 2011;22(4):1179–1189. 10.1353/hpu.2011.0127 22080702 PMC3586412

[4] Dickman SL , Gaffney A, McGregor A, et al Trends in health care use among black and white persons in the US, 1963-2019. JAMA Netw Open. 2022;5(6):e2217383. 10.1001/jamanetworkopen.2022.17383 35699954 PMC9198752

[5] Gong G , Phillips S, Hudson C, Curti D, Phillips B. Higher US rural mortality rates linked to socioeconomic status, physician shortages, and lack of health insurance. Health Affairs. 2019;38(12):2003–2010. 10.1377/hlthaff.2019.00722 31794316

[6] Ku BS , Druss BG. Associations between primary care provider shortage areas and county-level COVID-19 infection and mortality rates in the USA. J Gen Intern Med. 2020;35(11):3404–3405. 10.1007/s11606-020-06130-4 32827110 PMC7442285

[7] Ramesh T , Tsai TC. Hospital closures in rural communities of color: a double dose of inequality. J Rural Health. 2023;39(1):88–90. 10.1111/jrh.12704 35932091

[8] American Association of Medical Colleges . Physician specialty data report. Accessed November 10, 2022. https://www.aamc.org/data-reports/workforce/interactive-data/active-physicians-who-are-international-medical-graduates-imgs-specialty-2019

[9] Baer LD , Ricketts TC, Konrad TR, Mick SS. Do international medical graduates reduce rural physician shortages? Med Care. 1998;36(11):1534–1544. 10.1097/00005650-199811000-00003 9821941

[10] Thompson MJ , Hagopian A, Fordyce M, Hart LG. Do international medical graduates (IMGs) “fill the gap” in rural primary care in the United States? A national study. J Rural Health. 2009;25(2):124–134. 10.1111/j.1748-0361.2009.00208 19785577

[11] Tsugawa Y , Jena AB, Orav EJ, Jha AK. Quality of care delivered by general internists in US hospitals who graduated from foreign versus US medical schools: observational study. BMJ. 2017;356:j273. 10.1136/bmj.j273 28153977 PMC5415101

[12] Hagopian A , Thompson MJ, Kaltenbach E, Hart LG. The role of international medical graduates in America's small rural critical access hospitals. J Rural Health. 2004;20(1):52–58. 10.1111/j.1748-0361.2004.tb00007 14974436

[13] Al Ashry HS , Kaul V, Richards JB. The implications of the current visa system for foreign medical graduates during and after graduate medical education training. J Gen Intern Med. 2019;34(7):1337–1341. 10.1007/s11606-019-05027-1 31069706 PMC6614248

[14] Pomerantz RM . Applying for a sub-specialty fellowship: some tips and advice from a former program director. J Community Hosp Intern Med Perspect. 2011;1(3). 10.3402/jchimp.v1i3.8087 PMC3714037 23882331

[15] US Citizenship and Immigration Services . H and L filing fees for form I-129, petition for a nonimmigrant worker. US Department of Homeland Security. Accessed November 10, 2022. https://www.uscis.gov/forms/all-forms/h-and-l-filing-fees-for-form-i-129-petition-for-a-nonimmigrant-worker

[16] Tiako MJN , Fatola A, Nwadiuko J. Reported visa acceptance or sponsorship for non-US citizen applicants to US internal medicine residency programs. J Grad Med Educ. 2022;14(6):680–686. 10.4300/JGME-D-22-00072.1 36591431 PMC9765918

[17] American College of Physicians . The Role of International Medical Graduates in the US Physician Workforce. American College of Physicians; 2008.

[18] US Citizenship and Immigration Services . Conrad 30 waiver program. US Department of Homeland Security. Accessed November 10, 2022. https://www.uscis.gov/working-in-the-united-states/students-and-exchange-visitors/conrad-30-waiver-program

[19] Patterson DG , Keppel G, Skillman SM. Conrad 30 waivers for physicians on J-1 visas: state policies, practices, and perspectives. Final Report #157. Seattle, WA: WWAMI Rural Health Research Center, University of Washington; 2016.

[20] Aronovitz L . Preliminary Findings on the use of J-1 Visa Waivers to Practice in Underserved Areas. Government Accountability Office; 2006.

[21] PUBLIC LAW 103-416. October 25, 1994. https://www.govinfo.gov/content/pkg/STATUTE-108/pdf/STATUTE-108-Pg4305.pdf

[22] Patterson DG , Keppel G, Skillman SM, Berry C, Daniel C, Doescher MP. Recruitment of non-U.S. citizen physicians to rural and underserved areas through Conrad state 30 J-1 visa waiver programs. Final Report #148. Seattle, WA: WWAMI Rural Health Research Center, University of Washington; 2015.

[23] California Department of Health Care Access and Information . J-1 visa waiver program. Accessed on July 7, 2024. https://hcai.ca.gov/workforce/health-workforce/california-primary-care-office/j-1-visa-waiver-program/.

[24] Ramesh T , Brotherton SE, Wozniak GD, Yu H. Evaluation of the Conrad 30 waiver program's success in attracting international medical graduates to underserved areas. JAMA Health Forum. 2023;4(7):e232021. 10.1001/jamahealthforum.2023.2021 37505491 PMC10383001

[25] Health Resources and Services Administration . Designated health professional shortage areas statistics, first quarter of fiscal year 2022, designated HPSA quarterly summary as of December 31, 2021. Accessed November 5, 2023. https://data.hrsa.gov/Default/GenerateHPSAQuarterlyReport

[26] Baker O , Horvitz-Lennon M, Yu H. Racial and ethnic concordance between national health service corps clinicians and underserved populations. JAMA Netw Open. 2024;7(3):e242961. 10.1001/jamanetworkopen.2024.2961 38506809 PMC10955390

[27] Ramesh T , McBain RK, Cantor JH, Stein BD, Yu H. Mental health outcomes among patients living in US counties lacking broadband access and psychiatrists. JAMA Netw Open. 2023;6(9):e2333781. 10.1001/jamanetworkopen.2023.33781 37707819 PMC10502528

[28] Liu M , Wadhera RK. Primary care physician supply by county-level characteristics, 2010-2019. JAMA. 2022;328(19):1974–1977. 10.1001/jama.2022.15106 36378215 PMC9667327

[29] Ramesh T , Horvitz-Lennon M, Yu H. Opening the door wider to international medical graduates—the significance of a new Tennessee law. N Engl J Med. 2023;389(21):1925–1928. 10.1056/NEJMp2310001 37982423 PMC10688565

