# Correction to: An increasing number of states filled Conrad 30 waivers for recruiting international medical graduates

**DOI:** 10.1093/haschl/qxae121

**Published:** 2024-10-14

This is a correction to: Tarun Ramesh, Sarah E Brotherton, Gregory D Wozniak, Hao Yu, An increasing number of states filled Conrad 30 waivers for recruiting international medical graduates, *Health Affairs Scholar*, Volume 2, Issue 9, September 2024, qxae103, https://doi.org/10.1093/haschl/qxae103

In the version of this article published on 30 August 2024 an Acknowledgments section with details of funding support was inadvertently omitted. The article has been updated to include this section.

