# Supplementary material for: An increasing number of states filled Conrad 30 waivers for recruiting international medical graduates
Source: Health Aff Sch. 2024 Aug 19;2(9):qxae103. doi: 10.1093/haschl/qxae103 (PMC11364145; doi:10.1093/haschl/qxae103)
Supplement: qxae103_Supplementary_Data [file qxae103_supplementary_data.zip › Supplementary Materials.docx]

| Appendix Table 1: Distribution of Total Conrad Physicians by State | |  |  |
| --- | --- | --- | --- |
| State | **Total Conrad Physicians (2001-2020)** | **Proportion of Filled Slots (2001-2020)*** | **Proportion of Conrad Physicians in Rural Areas (2004-2020)** |
| Alabama | 339 | 57% | 24% |
| Alaska | 26 | 4% | 23% |
| Arizona | 557 | 94% | 35% |
| Arkansas | 356 | 60% | 33% |
| California | 537 | 91% | 67% |
| Colorado | 178 | 30% | 22% |
| Connecticut | 446 | 76% | 6% |
| Delaware | 321 | 54% | 18% |
| Washington, D.C. | 135 | 23% | 3% |
| Florida | 520 | 88% | 13% |
| Georgia | 462 | 78% | 26% |
| Hawaii | 87 | 15% | 31% |
| Idaho | 23 | 4% | 61% |
| Illinois | 579 | 98% | 17% |
| Indiana | 553 | 94% | 19% |
| Iowa | 579 | 98% | 36% |
| Kansas | 404 | 68% | 28% |
| Kentucky | 590 | 100% | 42% |
| Louisiana | 295 | 50% | 33% |
| Maine | 490 | 83% | 38% |
| Maryland | 477 | 81% | 43% |
| Massachusetts | 550 | 93% | 12% |
| Michigan | 590 | 100% | 37% |
| Minnesota | 499 | 85% | 41% |
| Mississippi | 167 | 28% | 51% |
| Missouri | 589 | 100% | 31% |
| Montana | 77 | 13% | 81% |
| Nebraska | 276 | 47% | 48% |
| Nevada | 221 | 37% | 13% |
| New Hampshire | 274 | 46% | 45% |
| New Jersey | 60 | 10% | 17% |
| New Mexico | 520 | 88% | 57% |
| New York | 590 | 100% | 21% |
| North Carolina | 289 | 49% | 70% |
| North Dakota | 207 | 35% | 16% |
| Ohio | 469 | 79% | 34% |
| Oklahoma | 265 | 45% | 44% |
| Oregon | 441 | 75% | 44% |
| Pennsylvania | 489 | 83% | 42% |
| Rhode Island | 421 | 71% | 0.2% |
| South Carolina | 453 | 77% | 31% |
| South Dakota | 180 | 31% | 31% |
| Tennessee | 282 | 48% | 33% |
| Texas | 551 | 93% | 18% |
| Utah | 101 | 17% | 6% |
| Vermont | 54 | 9% | 43% |
| Virginia | 432 | 73% | 47% |
| Washington | 555 | 94% | 37% |
| West Virginia | 411 | 70% | 43% |
| Wisconsin | 432 | 73% | 42% |
| Wyoming | 104 | 18% | 21% |
| Total | 18504 | 61% | 33% |
|  |  |  |  |
| Note: * Each state and Washington D.C., was allowed to have 590 slots in 2001-2020. The proportion of filled slots was calculated as the number of filled slots divided by 590 for each state and Washington D.C. in 2001-2020. | | | |
